# Supplementary material for: Computational analysis of congenital heart disease associated SNPs: unveiling their impact on the gene regulatory system
Source: BMC Genomics. 2025 Jan 21;26:55. doi: 10.1186/s12864-025-11232-6 (PMC11749323; doi:10.1186/s12864-025-11232-6)
Supplement: Supplementary file 1 — Supplementary Material 1. [file 12864_2025_11232_MOESM1_ESM.pdf]

Supplementary Figures

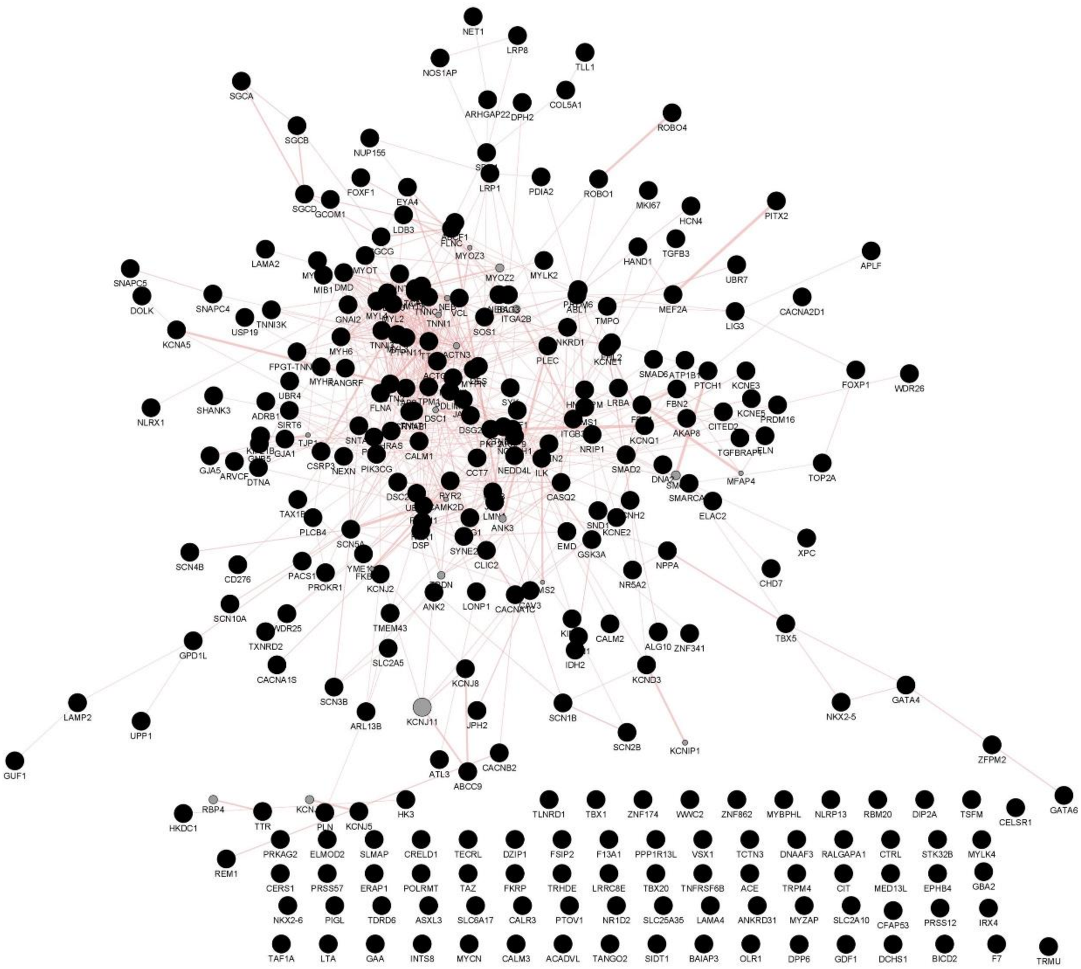

Figure S1. Protein-Protein interaction network (PPIN). In the PPIN, *nodes* represent proteins containing coding CHD-SNPs and the *edges* signify interactions between physically interacting proteins. The '*black'* nodes correspond to query proteins and '*grey'* nodes represent additional proteins in the network identified in the GeneMANIA query search. The size of each *'grey'* node is determined by their scores, reflecting the relatedness of these proteins based on GeneMANIA. Larger circles indicate a stronger predicted interaction between these proteins. *Width* of edges signifies the weight of interaction between proteins. Thicker edges indicate stronger physical interactions. The network is constructed using Cytoscape.

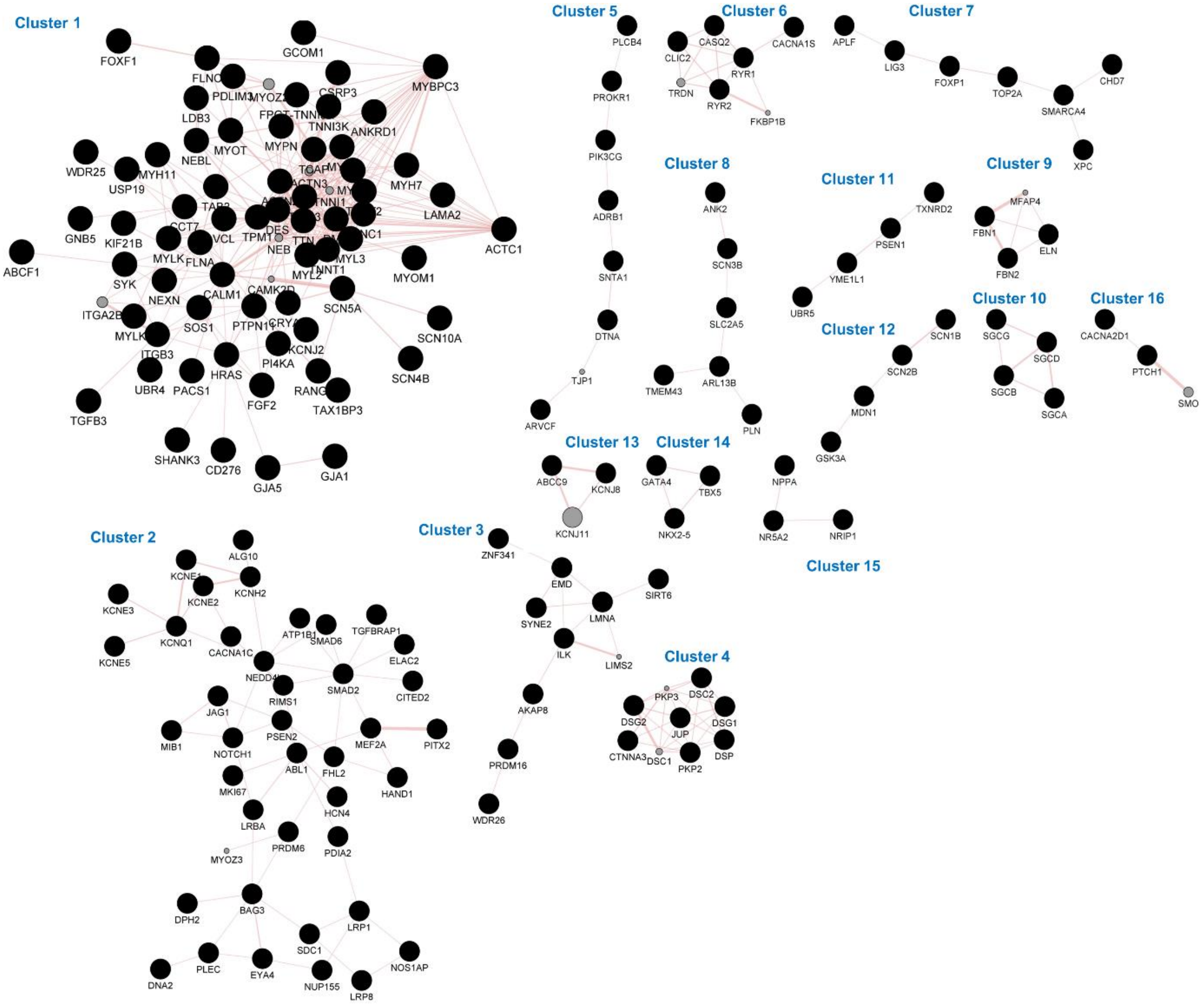

Figure S2. Clustering of PPIN using MCL algorithm.

PPIN comprises 16 distinct clusters numbered from 1 through 16 where *nodes* represent proteins containing coding CHD-SNPs and the *edges* signify interactions between physically interacting proteins.
